# An FPGA-Based High-Performance Stateful Packet Processing Method

**DOI:** 10.3390/mi14112074

**Published:** 2023-11-08

**Authors:** Rui Lu, Zhichuan Guo

**Affiliations:** 1 National Network New Media Engineering Research Center, Institute of Acoustics, Chinese Academy of Sciences, No. 21, North Fourth Ring Road, Haidian District, Beijing 100190, China; lur@dsp.ac.cn; 2 School of Electronic, Electrical and Communication Engineering, University of Chinese Academy of Sciences, No. 19(A), Yuquan Road, Shijingshan District, Beijing 100049, China; 3 Suzhou Haiwang Network Technologies Co., Ltd., Suzhou 215163, China

**Keywords:** FPGA, stateful data plane, configurable, PHV dynamic scheduling

## Abstract

Compared to a stateless data plane, a stateful data plane offloads part of state information and control logic from a controller to a data plane to reduce communication overhead and improve packet processing efficiency. However, existing methods for implementing stateful data planes face challenges, particularly maintaining state consistency during packet processing and improving throughput performance. This paper presents a high-performance, FPGA (Field Programmable Gate Array)-based stateful packet processing approach, which addresses these challenges utilizing the PHV (Packet Header Vector) dynamic scheduling technique to ensure flow state consistency. Our experiments demonstrate that the proposed method could operate at 200 MHz while adding 3–12 microseconds latency. The method we proposed also provides a considerable degree of programmability.

## 1. Introduction

### 1.1. Motivations

To face the emerging demands of network applications such as load balancing, TCP link tracing and stateful firewall [1,2], the control and management of network devices have become more complex. SDN (Software Defined Network) was proposed to address these issues by decoupling the control plane and the data plane, enabling centralized control of the network state. Furthermore, it provided excellent programmability and network control capabilities. OpenFlow [3] was the first implementation of the SDN protocol. Moreover, it offered program language and introduced the concept of flow tables, the “match-action” paradigm, which were used in the data plane of SDN architecture. The data forwarding plane only determined the forwarding behavior of packets based on the flow tables. The controller managed the corresponding flow tables on the data plane through the OpenFlow interface to control the forwarding behavior. This programming model supports stateful network functionality by executing stateful portions of the program on the controller and adjusting the packet processing rules accordingly. However, implementing simple stateful network applications such as TCP tracking and Round Trip Time estimation becomes impractical due to the requirement of back-and-forth packet transmission latency and overhead between the controller and the data plane.

The emergence of the stateful data plane has addressed these issues. It adopted the “match-state-action” paradigm, which offloaded a portion of the controller’s logic and state information to the data plane. This reduced the communications overhead between the controller and the data plane and improved the packet processing speed. The data plane chose appropriate packet forwarding strategies based on the state information. However, the stateful data plane increased the hardware complexity of network devices and led to many new challenges in the designing and management of the stateful data plane.

### 1.2. Limitations of Prior Art

Significant progress has been made in the implementation methods of stateful data planes, such as P4 [4,5,6], OpenState [7], Domino [8] and FlowBlaze [9,10]. However, current data plane methods face challenges in maintaining flow state consistency during high-speed packet processing. In this article, packets match the same flow table entry belonging to the same flow, and the flow state is shared among these packets. Therefore, the current hardware implementation of the stateful data plane involves frequent and continuous read, update, and write-back operations above the flow state memory. Hence, the consistency of the flow state is essential to ensure the correct behavior of the data plane. It was considered that PHV access to the same flow entry may have data dependency, and there is a certain clock cycle interval between the flow state read and write-back operation when implemented in hardware. The read operation may not obtain the latest flow state from the memory, which results in a RAW (Read After Write) hazard (true dependency). There is a need for a hardware-based method to schedule the packets processing sequence, allowing real-time detection of RAW hazards and utilizing hardware mechanisms to avoid RAW hazards while minimizing hardware pipeline stalls. However, the current methods [9,11,12,13,14,15] can not meet these requirements simultaneously. OPP proposed a hardware implementable state data plane architecture, but it did not consider the consistency of the flow state, which has potential risks. FlowBlaze introduced a simple round-robin PHV scheduler on the basis of OPP to ensure the consistency of flow state, but there is still room for performance improvement.

### 1.3. Proposed Approach

In response to the mentioned challenges, we proposed an improved hardware design that can be implemented on FPGA to support programmability in the data plane. Our proposed approach could dynamically schedule the processing sequence of PHV based on the data dependency of flow states, enabling the data plane to achieve flow state consistency while minimizing hardware pipeline stalls. We also propose an optional reorder buffer that could ensure the output order of PHV is the same as the input order. Consequently, subsequent modules will not be affected by out-of-order executions. Additionally, a series of RISC-like instruction sets and corresponding hardware structures are designed to enhance programmability and provide support for out-of-order executions. The main contributions of this paper include:

1. A match-action table that supports both stateful and stateless packet processing and can process 200 M PHV per second.
2. A series of RISC-like instruction sets and corresponding hardware structure designed based on basic packet processing requirements.
3. A hardware implementation method for PHV dynamic scheduling that is capable of achieving high-performance packet processing, flow state consistency, and maintaining the sequence of PHV input and output.

## 2. Related Works

OpenFlow [3] was the first meaningful implementation of the SDN paradigm. OpenFlow greatly enhanced the programmability of network forwarding, but its simple “match-action” abstraction does not support the management and utilization of stateful information in the data plane. Therefore, OpenFlow did not support stateful network applications, such as stateful firewalls.

OpenState [7] added a state management module to support the state management function on the basis of OpenFlow. OpenState offloaded a portion of the centralized controller logic to the data plane, and the forwarding behavior could be determined based on the local state information of the data plane. OpenState introduces the Extend Finite State Machine (EFSM) model to support programmability. The EFSM model uses the 4-tuple: state, input event, output and state transition to determine packet processing and state update behavior. However, the limitations of OpenState lie in that the programmability flexibility and expression of the basic EFSM model are relatively limited.

OPP (Open Packet Processor) [16] proposed a hardware-implementable eXtended Finite State Machine (XFSM) model, which introduced a condition evaluation process abstract that significantly reduced the size of a match table. OPP stored state information in the form of registers and used parallel processing modules composed of multiple ALUs to perform PHV actions and state updates, providing enhanced programmability. However, OPP did not consider the state’s inconsistent problem.

FlowBlaze [10] was an improvement on OPP. It introduced a round-robin PHV scheduler to avoid a state inconsistent problem. It also leveraged flow-level parallelism to improve packet processing speed. This approach explored the issue of consistency in the stateful data plane, but it still failed to meet the performance loss caused by flow state RAW hazards that need to stall the pipeline.

SDPA [17] proposed a different structure compared to the EFSM model. SDPA utilized the forwarding processor unit, which consists of the state table, state transition table and action table. This approach supported the simultaneous processing of multiple applications on the data plane. However, a drawback of SDPA was its implementation complexity and large match table size.

P4 [4] and Domino [18] are stateful data plane programming frameworks that use high-level abstraction languages to compile and implement stateful data planes. Both approaches used the directed acyclic graph to express the state machine. P4 utilized special registers to store the per-flow or global state information. Moreover, Domino transformed the packet process into a series of code blocks composed of simple operations and restricted the state-sharing mechanism between different processing units to guarantee data consistency.

In summary, many related works have been proposed and implemented in a wide range of stateful network applications using hardware structures or high-level abstraction languages as platforms. Representative examples of stateful data plane applications include port knocking, stateful firewalls and TCP link tracing. These applications leverage the characteristics of the stateful data plane to reduce communication with the controller and improve data plane processing speed [19]. They also utilized the programmability and flexibility of the stateful data plane to implement meaningful network functions. However, there are still challenges in flow state consistency when processing packets at full rate in the data plane. In comparison to OPP [16], our hardware architecture ensures flow state consistency. Compared to FlowBlaze [10], our approach demonstrated higher packet processing capabilities.

## 3. System Design

**Figure 1:**
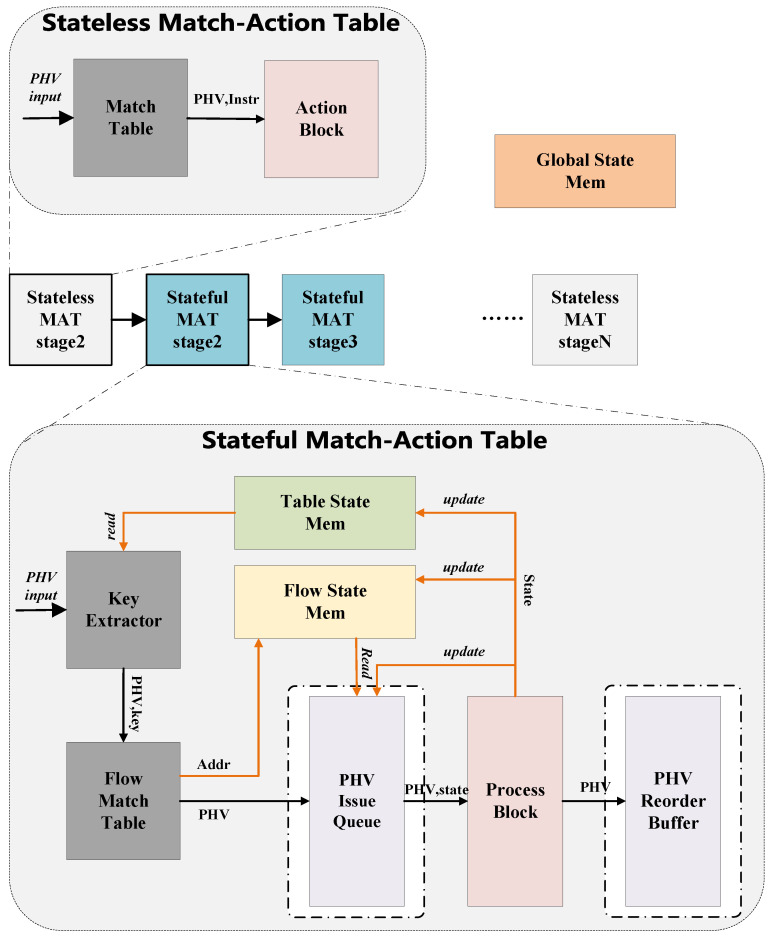
System architecture.

We propose an improved stateful data plane based on FPGA. Figure 1 shows its architecture, consisting of a series of stateful or stateless match-action tables. After the pre-processing of the parser module, the packets will enter the data plane pipeline in the form of PHV. The above-mentioned multi-level match-action tables will be configured with the corresponding instructions and flow entries. To achieve complex data plane functionalities, the fundamental function of each stage’s match-action table is storing and managing state information. By leveraging state information and the PHV, the modules perform match operations. Then, the operations of state updates and PHV modifications will be determined based on the match results.

### 3.1. System Overview

The overall architecture consists of hardware modules for storing and managing state information. The aforementioned storage module needs to solve the RAW hazard caused by frequent read/write operations and data dependency. Additionally, the consistency of flow states is the most important, as it determines the forwarding behavior of the data plane. The indiscriminate strong consistency guarantees for all state information will lead to a significant performance decrease in the packet process and lead to complex logic. Inspired by the article [20], we classified the states and adopted an abstract structure of hierarchical state domains. The hierarchical state domains consist of four levels: global, table, flow, and per-packet states. In our proposed architecture, the data plane contains global state memory, table state memory, and flow state memory. PHV contains packet states that pass information between different match-action tables. The definitions of the four levels of states are as follows:

- The global state is shared by all packets entering the switch.
- The table state is shared by packets entering the same match-action table.
- The flow state is shared by packets matching the same table entry.
- The per-packet state is used by an individual data packet to pass information between different match-action tables.

In our implementation, the state information is associated with the address as a unique identifier. This facilitates management and improves lookup speed. Considering the trade-off between performance and complexity of the design, the consistency of flow states is implemented by hardware, and the management of global and table states is handled by the controller.

The stateful match-action tables served as the fundamental building blocks of the stateful data plane. It has the following procedural sequence: by taking into consideration the input PHV, global state, table state and packet state, a unique key is generated. This key will be used in the match table and obtain the address of the PHV’s corresponding flow state. The flow state information is then attached by the address and fed into the issue queue. The issue queue schedules the PHV based on the dependency of the flow states, allowing out-of-order execution in the process block pipeline. The process block determines the behavior of state updates and PHV actions based on the configuration of the EFSM model abstract. The executed PHV will be stored in the reorder buffer and committed in order. The key differences between our work and previous research will be explained in the following subsection:

1. In Section 3.2, we describe the hardware implementation of PHV dynamic scheduling.
2. In Section 3.3, we describe the PHV process block that supports VLIW (Very Long Instruction Word) and its corresponding hardware structure.

### 3.2. PHV Out-of-Order Scheduling

**Figure 2:**
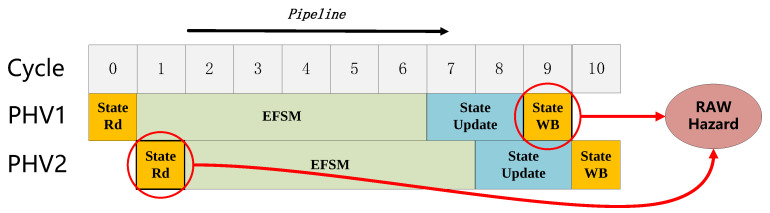
RAW hazard example.

The workflow of the stateful data plane can be summarized as read, update and write-back operations of the flow state. Therefore, the consistency of read after write operations is essential in ensuring behavior correctness. However, read and write operations occur nearly every clock cycle due to high-speed packet processing. Flow state read operation may not obtain the latest data from the flow state memory, resulting in the RAW hazards (true dependencies). This can occur because even though a PHV action is executed after a prior PHV action, the prior PHV action has been processed only partly through the pipeline. Figure 2 provides an example of PHV out-of-order scheduling. PHV1 and PHV2 are two PHVs of the same flow, and the flow state read operation of PHV2 happened before the updated flow state of PHV1 write-back, resulting in the RAW hazard. Additionally, speculation techniques are infeasible; PHV2 needs to be blocked until the update flow state of PHV1 is already written back. However, stalling the pipeline will significantly decrease performance. Considering the potential parallelism that can be utilized from the PHV of distinct flows, we proposed a dynamic scheduling method. This method will detect the data dependency and dispatch the PHV and its corresponding state to the process block pipeline out-of-order, thereby enhancing the speed of packet processing.

**Figure 3:**
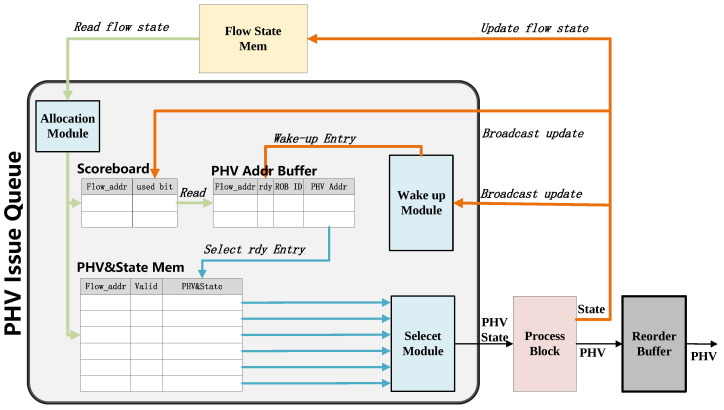
Implementation of PHV dynamic scheduling.

However, the out-of-order executions may affect subsequent modules. To address this problem, an optional hardware-based solution has been proposed to ensure flow state consistency in high-speed packet processing, which includes the PHV issue queue and PHV reorder buffer modules. As shown in Figure 3, the PHV issue queue together with the reorder buffer consists of our dynamic scheduling method. PHV would obtain its corresponding flow state from the memory module or process block’s broadcast. Our PHV issue queue module allows real-time detection of RAW hazards and accordingly dispatches the PHV out-of-order. Moreover, it utilizes hardware mechanisms to avoid errors while minimizing hardware pipeline stalls. On the other hand, the PHV reorder buffer module will reorder the executed PHV to avoid the effects of out-of-order executions.

#### 3.2.1. PHV Issue Queue

In our proposed PHV issue queue, PHV and the corresponding state information can be considered as a combination of instructions and data. They will be dispatched to the process block pipeline for subsequent “match-action” execution. The issue queue will detect real-time RAW hazards between the input PHV and change the execution sequence of PHV to avoid hazards and reduce pipeline stalls. Similarly, the function of the aforementioned module is similar to the instruction issue queue in modern processor architecture [21,22]. As mentioned in Section 3.1, the hierarchical state storage mechanism utilizes an address as an identifier for state information. The above addresses can be used for reading and writing back the latest flow state. Therefore, the issue queue can detect the PHV’s corresponding flow state dependencies based on their unique address. Moreover, the issue queue scheduling range is defined as the out-of-order window, where the window size is equal to the depth of reorder buffer. Our proposed PHV issue queue is composed of the following four modules (recall Figure 3):

- Allocation Module: This module finds the free entry with the lowest address in the PHV Address Buffer and the free entry of PHV&State Mem. If no free entry is left, it will block the input data stream.
- Scoreboard: This module is a memory of flip flops that stores the status of the per-flow state and keeps track of per-flow state read and write operations. So, the number of flip flops is equal to the number of the flows. The scoreboard will be updated by the broadcast buses at every positive edge of the clock. So, this module is capable of detecting RAW hazards between the PHV’s corresponding flow states. The fields of used bits in the scoreboard indicate the validity of the input PHV’s flow state. Every time a new PHV enters, the system will check if the used bit is busy or not and read in the corresponding flow state. This module also checks the broadcast buses if there is a conflict in broadcast line and reads the line of the same flow address.
- PHV&State Mem: This module stores input PHV and its corresponding state information in the address of the memory given by the Allocation Module.
- PHV Addr Buffer: This module stores the address of the input PHV and its corresponding state in the lowest address free entry of the buffer given by the Allocation Module. Every entry includes the following fields: PHV&flow addr together with its ready bit (used to indicate whether the PHV has a dependency relationship), ROB (Reorder Buffer) ID. To maintain the old-first order in this buffer, bubble entry resulting from out-of-order dispatch needs to be considered. By shifting down the entries above the bubble entry, the issue queue could maintain the old-first rule. Additionally, this module associates an increasing ROB ID to each input PHV within the out-of-order window (size equal to the reorder buffer), which is used for PHV reordering.
- Select Module: In each clock cycle, the Select module will choose the lowest-address valid entry in the PHV Addr buffer, which is marked as ready to be dispatched. Then, this module dispatches the selected entry to the subsequent process block pipeline.
- Wake-up Module: This module continuously monitors the broadcast buses. Whenever the executed flow state address broadcasted matches the PHV Addr buffer’s entry, the corresponding flow state will be updated and marked as ready. If multiple entries match the broadcasted address, only the oldest one will be chosen as ready.

The core workflow of the PHV issue queue can be summarized as following two parts: First, based on the Scoreboard, potential RAW hazards between PHVs are detected. PHV that are not ready will be blocked, while allowing the irrelevant PHV to execute ahead. The wake-up module monitors the broadcast and activates the oldest PHV entries that have data dependencies. So, this nodule ensures that PHV of the same flow are dispatched in order and avoid RAW hazards, while allowing out-of-order launch for different flow PHVs. Second, the issue queue stores the input PHV and their corresponding states. Each cycle, ready PHV will be selected for dispatch into the pipeline, and the empty bubbles are eliminated by shifting operations. Below, there will be detailed explanations and figures about the mentioned workflow provided.

**Figure 4:**
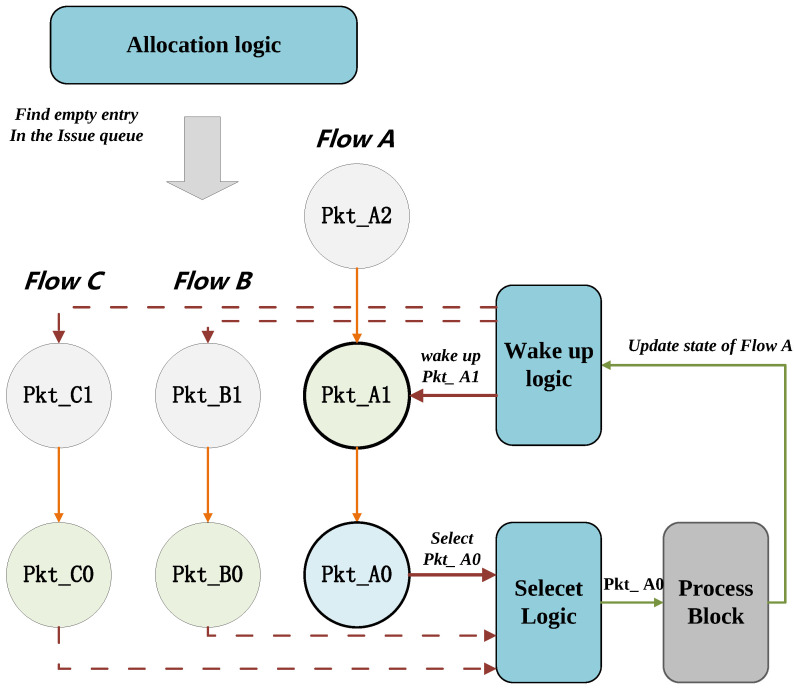
Out-of-order scheduling workflow.

In Figure 4, the arrow indicates the data dependency of the packets. In the beginning, only the packets at the bottom of the buffer are ready to be dispatched. There are seven packets of three different flows in the issue queue waiting to be dispatched. Currently, only packets A0, B0 and C0 are ready, with no data dependencies. Other packets need to wait until the latest results of the flow states they require are already written back. For example, packet A1 must wait until packet A0 is executed and broadcasted to the wake-up module, and then, its flow state becomes ready. Similarly, packet A2 needs to wait for the execution result of packet A1 to be written back. In this example, packet A0 is the first to be dispatched into the pipeline, but its flow state updated result needs to wait for several cycles before it is broadcasted. During this waiting period, pkt B0 and pkt C0 will be dispatched in turn.

**Figure 5:**
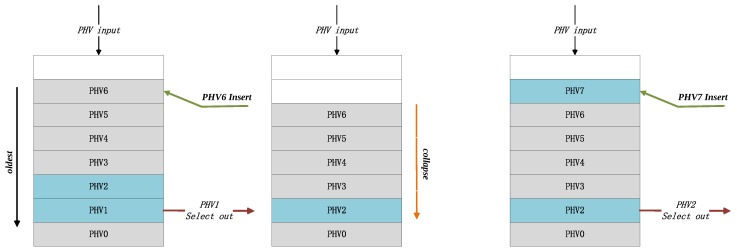
PHV issue queue example.

In Figure 5, for each clock cycle, a PHV in the buffer will be dispatched if it is ready, and a new input PHV will be inserted into the buffer if valid. We illustrate the typical workflow of the PHV issue queue: to ensure in-order execution of the same flow, the PHV Addr buffer’s entries must strictly follow the old-first order rule. Therefore, issue queue has to collapse the entry in the PHV Addr buffer when there is out-of-order dispatching (e.g., PHV1 dispatched). However, if the buffer stores both the PHV and state information with large bit widths, collapsing the entries in hardware implementation means significant resource and energy consumption. Therefore, we improved our method by using second-level address mapping. So, the PHV ADDR buffer’s entries only store the address of the PHV and state. The collapse operation is performed on the addresses rather than the PHV data. The actual data information is stored in an additional RAM module named PHV&State Mem.

#### 3.2.2. Reorder Buffer

**Figure 6:**
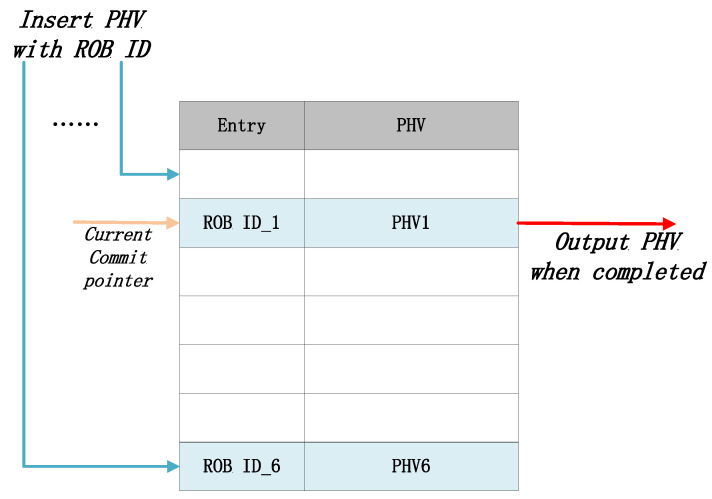
Reorder buffer.

The function of the Reorder Buffer is to store out-of-order executed PHVs and commit them in order. In our implementation, shown in Figure 6, the ROB structure is realized as a circular buffer and this module is optional on demand. When a PHV finishes its execution in the pipeline, it will be written into the corresponding entry of the circular buffer according to its ROB ID allocated by the issue queue. Simultaneously, the commit pointer starts from the base address and waits for the pointed entry’s PHV to be written back or currently ready, then commits this entry’s PHV. Once the PHV is committed, the pointer’s address is incremented and these operations are continued. The width of buffer equals the length of PHV, and the depth of FIFO equals the out-of-order window size. The out-of-order window was the maximum out-of-order scheduling range allowed by the issue queue. This out-of-order window ensures that the PHV’s write addresses do not conflict in ROB. We choose the exponent of 2 as an optional parameter, for example, 16, 32, 64. In addition, the size of the out-of-order window needs to be greater than the issue queue depth. However, ROB at 64 depth takes up too much hardware resources and does not improve performance. In summary, the size of out-of-order window could be 16 or 32, depending on the parameters of the issue queue. The reorder buffer ensures that the PHVs are committed in the sequence as their arrival sequence.

#### 3.2.3. Necessity of Old-First Rule

Our approach chose to collapse the bubble entry in the issue queue and restrict the PHV in old-first order. This brought advantages that simplify the Select Module, as the PHV are arranged from top to bottom in descending order of their existing time, making it easy to use a priority encoder to select the oldest PHV that is ready to be executed. The Allocation module is also simplified by keeping the free entries in the upper region of the buffer. Additionally, the depth of the issue queue together with the size of the out-of-order window will determine the capabilities of dynamic scheduling. However, the issue queue’s depth bottleneck lies in the Wake-up module and the Select module’s hardware structure. The latency of the mentioned module exhibits a linear correlation with the depth of the issue queue, which means the larger the number of entries the issue queue contains, the higher the latency is. Therefore, it is necessary to decide on the issue queue parameters based on actual deployment requirements and the execution cycle of the process block needed. In Section 4.1, we discuss the trade-off between the resources and performances of the issue queue based on practical experience.

### 3.3. PHV Process Block

**Figure 7:**
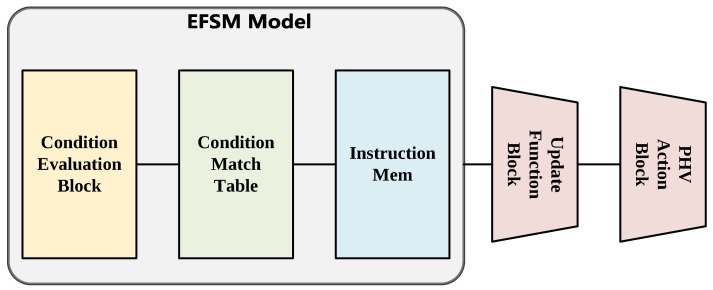
Overview of the process block.

This section will introduce the design of the process block, which is the core of PHV modification and state update. The ready PHV and corresponding state information are dispatched into the process block via the issue queue, and the pipeline uses the EFSM (Extend Finite State Machine) abstraction to decide the forwarding strategies, as shown in Figure 7. In our implementation of the process block, when the PHV is dispatched into the pipeline, 8 cycles are required for the completed state update, and 10 cycles are required for the completed PHV action. Moreover, the execution clock cycles are also affected by the size of the TCAM (Ternary Content Addressable Memory) match table in the EFSM model. The processing steps of this pipeline are briefly described as follows:

1. EFSM Model: Based on the configured VLIW, the EFSM model calculates the vector-form condition evaluation results. The condition results and state information are used as input for the EFSM table, which could determine the address of the corresponding instructions for the processing strategy. The instructions will be fetched from the instruction RAM.
2. State Update Block: Based on the input VLIW, the state update block performs the necessary operations to update the state.
3. PHV Action Block: Based on the input VLIW, the PHV action block performs the necessary operations to modify the PHV.

**Figure 8:**
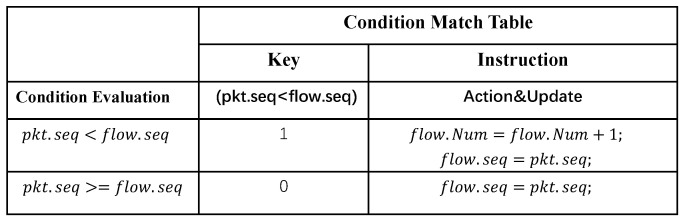
EFSM Module.

The PHV action and state update instruction are determined based on the match results of the EFSM model. Algorithm 1 shows a simple application running on a stateful data plane. It will count the number of TCP retransmission packets per flow, based on the size relationship between per flow sequence and packet sequence. Figure 8 explains how the EFSM model works, how to determine the packet forwarding strategy using PHV and state information and shows the hardware implementation abstraction. The condition evaluation block calculates the comparison result (pkt.seq < flow.seq). The condition match table uses comparison results as a match key to decide the state update instruction based on the match results (address of instruction). Moreover, in the state update block, the flow state (flow.seq, flow.num) would be updated according to the instruction obtained in the instruction memory module.
**Algorithm 1**: Calculate the Number of TCP Retransmission PacketsINPUT:TCPpacketpkt$OUTPUT:TCP\;packet\;pkt^{\prime }$**if** pkt.seq<flow.seq **then**   flow.num=flow.num+1**end if**flow.seq=pkt.seq

#### 3.3.1. Structure

**Figure 9:**
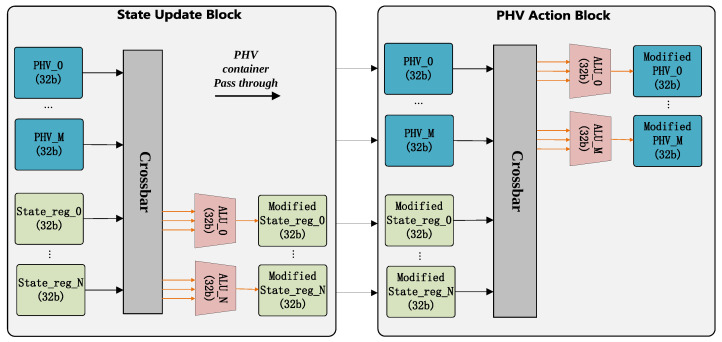
ALU block.

To enhance programmability and flexibility, the data plane supports extracting arbitrary fields from the PHV and state registers according to the instructions, as shown in Figure 9. The ALU block consists of two modules: state update block and PHV action block. The structure of these two modules is similar but are responsible for different things. Each module is fully pipelined and needs two cycles to complete its task. In the first cycle, the crossbar module could obtain the input register according to the instructions’ rs information. In the second cycle, the calculation process is completed by several parallel ALUs, and the number of ALUs is equal to the width of the PHV or state. Each ALU supports arithmetic operations, comparisons and bit manipulation with 32/16/8-bit register operand or immediate operand. The results are written back to the corresponding container for each simple ALU. In the ALU block, the PHV and state information are divided into containers, with each container holding 32-bit data. Since the maximum number of registers in a RISC-like instruction set is 32, Action Block supports PHV and state information with a maximum width of 1024 bits. The number of ALU in the state update block and PHV modification block is directly related to the required data width for processing. To reduce the crossbar resource usage, the crossbar module does not support arbitrary writing back data paths specified by the instructions. Instead, the results are written back to the corresponding container for each ALU. To reduce the state update clock cycles, the state update block is placed before the PHV action block.

#### 3.3.2. VLIW

In Figure 9, for the purpose of providing better data plane programmability, condition evaluation block, state update block and PHV action block support VLIW as the input instructions. Those modules consist of multiple parallel ALUs, using an instruction set similar to RISC-V. Additionally, they support the encapsulation of multiple independent instructions into a single long instruction, which is then submitted to multiple vectorized ALUs for parallel processing and outputs the results. Each ALU supports arithmetic operations, comparisons and bit manipulation with 32/16/8-bit register operand or immediate operand. It also supports selecting relative input and output data addresses (e.g., result.B3 = oprand0.B0 + oprand1.B2, where the instruction can specify the input data addresses and the output data position). Furthermore, to meet the basic requirements of packet processing algorithms, a set of 32-bit RISC-like fundamental instructions have been designed to enhance programmability.

## 4. Hardware Implementation

Based on the proposed high-throughput data plane architecture above, we implemented a multi-stage stateful match-action table pipeline deployed on the Xilinx Zynq UltraScale+ FPGA chip on a Dell R740 server. Furthermore, we downloaded the generated bit file to the FPGA device. To generate test data streams, we utilized the IXIA high-speed network traffic generation tool and configured the stateful data plane as a simple Multi-Path network application. Limited by data bus width and the parser module, the PHV processing speed of the pipeline has redundancy, especially when dealing with a packet of large size. In our deployed multi-path applications, the data plane pipeline will not drop the packet. The packet loss only occurs in the FIFO used as the interface between different modules. The pipeline processing speed could not reach 200 Mpps due to bottlenecks in other modules such as the parser, deparser and data bus width. We verified this 200 Mpps capability in simulation software.

### 4.1. Hardware Resources

Our specific parameters for hardware implementation are shown in Table 1. The flow match table and flow state memory utilize BRAM resources. Each entry above supports a maximum key size of 256 bits (supporting two combined 128-bit IPv6 addresses) and 128 bits of flow state information (corresponding to four containers). Due to the resources and time limitations, the size of the EFSM table is relatively small, with each table entry fixed at 40 bits (8 bits for the condition evaluation results and 32 bits for the state information) and 16 entries implemented as a TCAM table. Our approach supports a maximum of 704 bits for PHV fields, 64 bits for global state fields, 64 bits for table state fields, 64 bits for packet state fields, and 128 bits for flow state fields. These total 1024-bit fields correspond to 32 containers.

With theoretical analysis and experiment, we evaluated the specific resource and module parameters of the implementation. Table 2 lists the FPGA luts and flip flops the resource utilization of the hardware implementation. In comparison, the resource requirements of FlowBlaze’s single-stage match-action element are also included. The listed resources only include the resource overhead for the stateful data plane and do not consider the hardware resource overhead of the network interface framework based on Corundum [23].

Moreover, we use the same algorithms to compare our work and software implementation method. Forwarding latency is used as a performance comparison metric between hardware implementation and software implementation. The application was a simple stateful forwarding, and the packet’s outport was determined based on the flow state information. The stateful data plane dynamically determines whether to send packets to outport 0 or outport 1 by polling its current state. As shown in Table 3, even for simple applications, hardware forwarding latency still has significant advantages over software implementation.

### 4.2. Issue Queue Parameters Analysis

**Figure 10:**
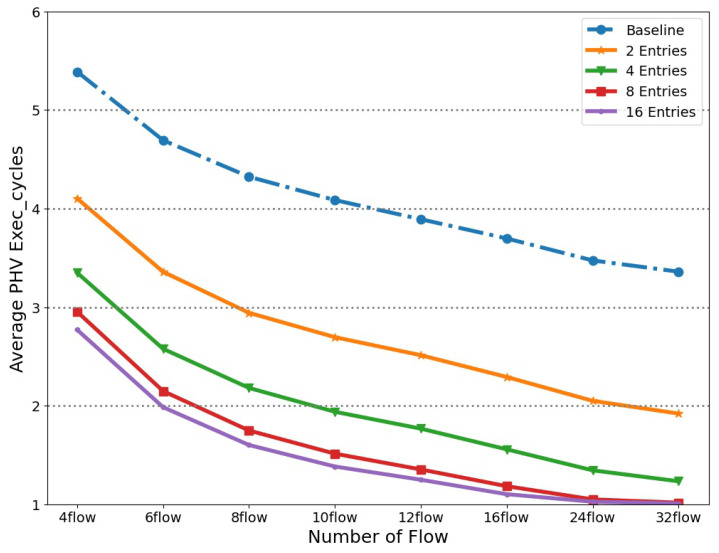
Issue queue performance with different depths.

In this section, the issue queue’s depth (number of entries) parameters will be discussed. This parameter relates to PHV processing speed and the corresponding resource utilization in typical scenarios. Based on the experiment and analysis, we provide appropriate issue queue deployment parameters. Firstly, we define that the input packets processed by the current data plane belong to a certain number of equiprobable flows. Our pipeline design only supports stalling the pipeline and does not drop packets. The average execution cycle of PHV is used as the performance metric. The pipeline’s input was a continuous stream of 100 M packets of equiprobable 4, 6, 8, 10, 12, 16, 24 and 32 flows. We analyzed the relationship between the average PHV processing clock cycles and the depths of issue queue in different situations, as shown in Figure 10.

**Figure 11:**
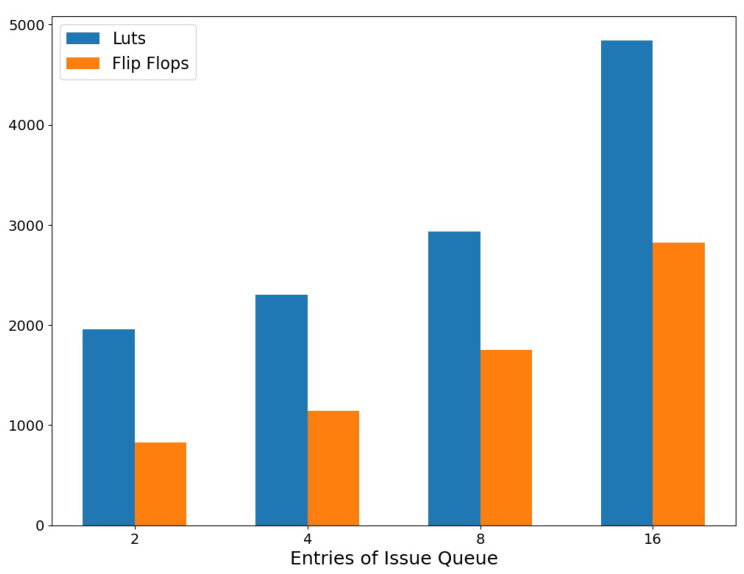
Issue queue resources.

The relationship between the number of issue queue entries and the FPGA hardware resources overhead is shown in Figure 11. It can be observed that hardware resource consumption has a linear relationship with the depth of the issue queue. Additionally, due to the characteristics of wake-up and select logic in the issue queue, the delay is positively correlated with the depth. Our practical experience has shown that issue queues with a depth over 16 will face more significant timing constraint problems when the overall FPGA hardware resource utilization is high. Recalling Figure 10, the performance of the issue queue is related to its depth. In typical scenarios with a 32 flow packets input, the issue queue with relatively larger depths can approach full speed. A larger depth implies stronger scheduling capabilities and higher performance, but there are diminishing marginal returns after eight entries. Considering the trade-off between resources and performance, the issue queue module chooses eight entries as the parameter.

### 4.3. Method Comparison

**Figure 12:**
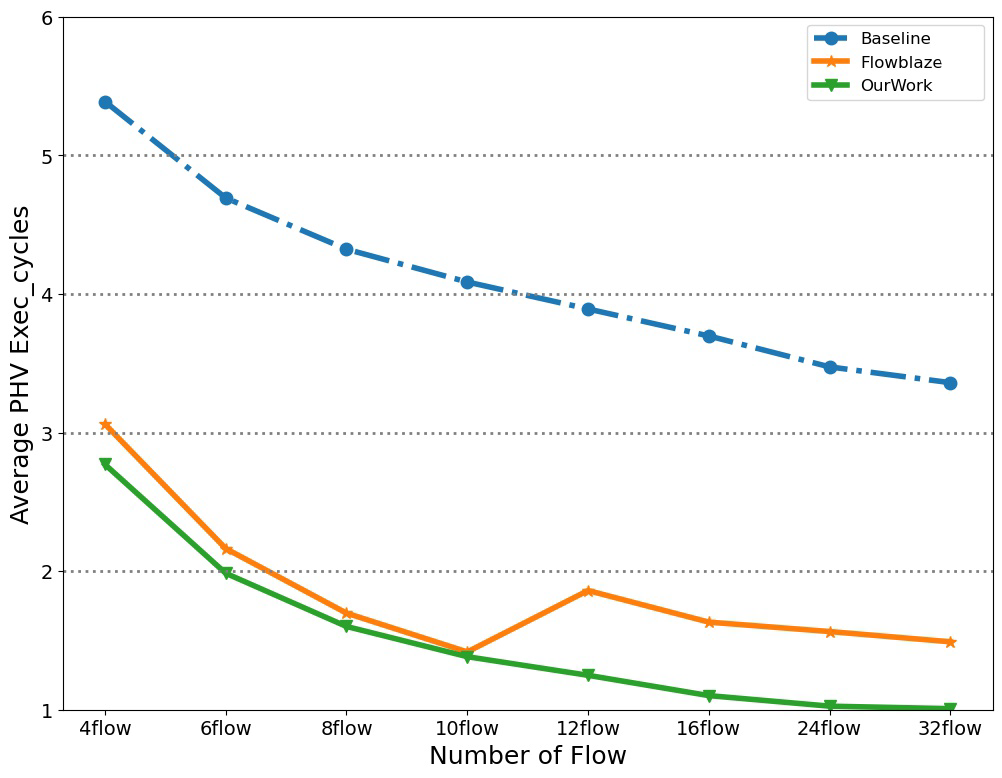
Performance comparison (FlowBlaze [10]).

In Figure 12, we compared the average PHV execution cycles of different methods. The “baseline” represents no out-of-order scheduling and only blocks the pipeline until the depend flow state is written back. It can be observed that the proposed PHV scheduling method in our work outperforms the PHV round-robin method in FlowBlaze [10] in typical scenarios. The reason is that when polling multiple queues, the round-robin method cannot guarantee that the PHV whose data is ready will be dispatched in time. Another reason is that our scheduling module was placed after the flow state read operations, which means less RAW stalling time overhead. Additionally, there is a bottleneck on the PHV average execution cycles in FlowBlaze, and this bottleneck can only be improved by increasing the queue capacity.

Due to the existence of up to 16 possible branches for updating the flow state, speculation techniques are difficult to implement in the stateful data plane. Therefore, considering only the delay metrics within the data plane pipeline, emitting in old-first order is the optimal choice. In our test, we configured the IXIA device to send packets at a rate of more than 80 Gbps to measure the throughput performance of our work. Table 4 provides a comparison of the data plane processing performance of various existing methods.

## 5. Conclusions

In this article, we propose a novel method for implementing stateful data planes, achieving a great improvement in data plane processing performance. Our proposed work could achieve a PHV processing speed of up to 200 M per second while adding 3–12 microseconds of latency. In addition, the PHV process capabilities per clock cycle have significantly improved compared with FlowBlaze [9]. The PHV issue queue module was introduced, using the dynamic scheduling technique to detect flow state dependency and effectively avoid RAW hazards. With the reorder buffer, the PHVs are committed in the same order as the input, avoiding the effects of out-of-order executions. Furthermore, a series of 32-bit RISC-like instructions set and corresponding hardware structure is designed based on the basic requirements of packet processing algorithms to support better programmability and network function extension. The hardware implementation method in this article is deployed on Xilinx Ultrascale+ FPGA, which could achieve high-performance packet processing while maintaining a good balance between resource consumption and performance. In summary, our method presents a novel and effective way for implementing stateful data planes, addressing the contradiction between high-performance packet processing and flow state consistency. It achieves better programmability and broader functionality support while demonstrating excellent performance in terms of hardware resource consumption and processing speed. The proposed approach also provides strong support for the deployment of high-performance network applications. 

## Figures and Tables

**Table 1:** Implement parameters.

Modules	Parameters	Description
Global state mem	64 b × 8	width and entry num
Table state mem	64 b × 8	width and entry num
Flow state mem	128 b × 4 K	width and entry num
Pkt state reg	64 b	width
Condition Block	8	ALU num
Update Block	10	ALU num
Action Block	22	ALU num
Issue Queue	8	entry num
Reorder Buffer	32	depth

**Table 2:** FPGA resource utilization.

	Our Work	FlowBlaze [10]
Luts	60,871 (12%)	71,712 (14%)
Flip Flops	35,898 (3%)	Unknown

**Table 3:** Simple forwarding latency.

	Our Work	Software [20]
latency/us	2.9	13.8

**Table 4:** Comparison of existing methods [1].

Platform	Hardware Storage	Throughput
Our Work	TCAM, BRAM, Register	80 Gbps
Openstate [7]	TCAM, RAM	Unkown
FlowBlaze [10]	TCAM, BRAM, Register	14.8 Mpps
SNAP [15]	CAM, Register	Unkown
Opp [16]	TCAM, RAM, Register	10–80 Mpps
SDPA [17]	TCAM, RAM	0.5–10 Gbps

## Data Availability

All the necessary data are included in the article.
